# In-Stent Restenosis Pathophysiology and Risk Factors: A Comprehensive Review

**DOI:** 10.3390/medicina62020345

**Published:** 2026-02-09

**Authors:** Alice Elena Munteanu, Alexandru Andrei Badea, Alexandru Mihai Popescu, Florentina Cristina Pleșa, Silviu Marcel Stanciu

**Affiliations:** 1 Department of Cardiology, ‘Carol Davila’ Central Military Emergency University Hospital, 010825 Bucharest, Romania; 2 Department of Medical-Surgical and Prophylactical Disciplines, Faculty of Medicine, ‘Titu Maiorescu’ University, 031593 Bucharest, Romania; 3 Doctoral School, Faculty of Medicine, “Carol Davila” University of Medicine and Pharmacy, 050474 Bucharest, Romania; 4 Department No. 6—Clinical Neurosciences, ‘Carol Davila’ University of Medicine and Pharmacy, 050474 Bucharest, Romania; 5 Department No. 5—Internal Medicine, ‘Carol Davila’ University of Medicine and Pharmacy, 050474 Bucharest, Romania

**Keywords:** in-stent restenosis, drug-eluting stent, bare-metal stent, percutaneous coronary intervention, neointimal hyperplasia, in-stent neoatherosclerosis, vascular smooth muscle cells, endothelial dysfunction, acute coronary syndrome, target lesion revascularization

## Abstract

In-stent restenosis (ISR) remains a clinically relevant cause of recurrent ischemia and repeat revascularization despite progressive refinements in stent design and implantation technique. Contemporary data indicate that restenosis-related target lesion revascularization (TLR) has declined from bare-metal stent (BMS) to early- and newer-generation drug-eluting stents (DESs), yet ISR continues to accumulate over long-term follow-up and is associated with worse outcomes than PCI for de novo lesions. Mechanistically, ISR is a time-dependent, heterogeneous process dominated early by neointimal hyperplasia—triggered by mechanical endothelial injury, delayed re-endothelialization, inflammation/oxidative stress, vascular smooth muscle cell phenotypic switching, and extracellular matrix deposition—and later by in-stent neoatherosclerosis, which may confer a higher-risk plaque substrate and overlap with thrombotic complications. Clinically, ISR frequently presents as an acute coronary syndrome (ACS) rather than stable symptoms, underscoring the prognostic relevance of prompt recognition and mechanism-informed management. Patient-level risk determinants repeatedly reported across cohorts include diabetes mellitus, chronic kidney disease, dyslipidemia, hypertension, and smoking, while lesion/procedural factors include small vessel caliber, long/complex or bifurcation lesions, multiple stent layers, and suboptimal stent expansion. Intravascular imaging (OCT/IVUS) is central to phenotyping ISR mechanisms (e.g., underexpansion, calcific neoatherosclerosis, stent fracture, homogeneous hyperplasia) and can guide targeted prevention and therapy. This review synthesizes current evidence on ISR biology and risk factors to support risk stratification, preventive strategies, and individualized management.

## 1. Introduction

Percutaneous coronary intervention (PCI) has fundamentally transformed the management of coronary artery disease by restoring coronary blood flow through intracoronary devices, predominantly contemporary second-generation drug-eluting stents (DES), which demonstrate superior safety and efficacy compared with bare-metal stents (BMS) and first-generation DES that are now largely obsolete in routine clinical practice.

Patients with prior PCI (typically with contemporary second-generation DES may experience recurrent chest pain for several reasons, including incomplete revascularization with residual coronary lesions, progression of native-vessel atherosclerosis, and in-stent restenosis (ISR). In addition, persistent or recurrent angina may reflect coronary vasomotor disorders (epicardial or microvascular spasm) and/or coronary microvascular dysfunction, which can be identified using invasive coronary function testing (CFT), including coronary flow reserve (CFR) assessment with pharmacologic hyperaemia (e.g., adenosine or intracoronary papaverine), indices of microvascular resistance, and acetylcholine provocation testing [1,2,3].

Despite advancements in stent technology and procedural techniques, ISR remains a major clinical challenge, contributing to recurrent symptoms and the need for repeat interventions. ISR is defined as the narrowing of the vascular lumen following PCI, with or without stent implantation. If a stent has not been implanted, the underlying mechanism is mainly vascular remodeling and elastic recoil. On the other hand, when a stent has been implanted, restenosis is caused either by neointimal proliferation or by neoatherosclerosis at that location [4].

The incidence of ISR varies based on multiple factors, including patient characteristics, stent type, lesion complexity, and procedural techniques [5,6]. While early-generation DES demonstrated a significant reduction in ISR rates compared to BMS, restenosis remains an issue, particularly in high-risk patients with comorbidities such as diabetes mellitus, chronic kidney disease, and dyslipidemia. Additionally, emerging evidence suggests that factors such as endothelial dysfunction, oxidative stress, and vascular inflammation play critical roles in the pathogenesis of ISR.

A review of randomized controlled trials (RCTs) revealed that the one-year incidence of target lesion revascularization (TLR) due to ischemia was 14.7% for BMS, 4.9% for early-generation DES, and 2.5% for newer-generation DES. The risk of ISR with BMS peaked during the first year. However, between years 1 and 5, the cumulative ischemia-driven TLR rates were 6.1%, 5.9%, and 4.4% for BMS, early-generation DES, and newer-generation DES, respectively [7].

Data from a large multicenter registry, which included over 48,000 de novo lesions treated between 2002 and 2016, showed that ISR rates increased after each reintervention. After the first, second, and third reinterventions, the ISR rates were 8.3%, 17.1%, and 22.8%, respectively [8].

A meta-analysis revealed that PCI for ISR is associated with a higher occurrence of adverse cardiac events compared to PCI for newly developed lesions [9].

This article aims to provide a comprehensive review of the risk factors associated with ISR, detailing the pathophysiological mechanisms.

## 2. Materials and Methods

### 2.1. Review Type and Objective

This manuscript is a narrative, non-systematic review intended to summarize contemporary mechanisms, imaging correlates, and management strategies for coronary ISR and neoatherosclerosis, with emphasis on current-generation DES and drug-coated balloons (DCB). The objective (stated in the Introduction) was to synthesize clinically actionable evidence while clearly distinguishing established evidence (guidelines, randomized trials, meta-analyses) from hypothesis-generating observational or mechanistic data.

### 2.2. Data Sources and Search Strategy

A structured but non-exhaustive literature search was performed in PubMed/MEDLINE and Embase, supplemented by the Cochrane Library for systematic reviews/meta-analyses and Google Scholar for citation-chaining of seminal papers and key intravascular imaging registries. Additional targeted searches were performed on major society guideline repositories to capture contemporary recommendations relevant to ISR, intravascular imaging, and antithrombotic therapy.

The search covered publications from 1 January 2000 to 31 December 2025, with the final search run on 24 January 2026. Evidence was prioritized using a pragmatic hierarchy: (i) international guidelines/consensus statements, (ii) randomized controlled trials, (iii) systematic reviews/meta-analyses, (iv) large registries and intravascular imaging cohorts, and (v) smaller observational studies and mechanistic/pathology reports (used primarily to support biological plausibility or device-healing concepts).

Records were screened at the title/abstract level and then in full text using predefined eligibility criteria, with disagreements resolved by consensus. Inclusion criteria were: adult human studies of coronary ISR, stent thrombosis, or neoatherosclerosis; studies evaluating mechanisms (clinical, pathology, or intravascular imaging) or treatment strategies (repeat DES, DCB, atherectomy, intravascular lithotripsy where applicable); guideline documents relevant to PCI, intravascular imaging, and antithrombotic therapy; and studies reporting clearly defined endpoints and/or imaging definitions. Exclusion criteria were: non-coronary stent literature, non-human studies (unless essential for mechanistic context and explicitly labeled), conference abstracts without full data, duplicate publications, and studies without sufficient methodological detail to interpret definitions (e.g., unclear ISR/neoatherosclerosis criteria).

### 2.3. Data Extraction and Synthesis

For included studies, the following data were extracted when available: study design, sample size, clinical setting (stable CAD vs. ACS), device type (BMS, first-/second-generation DES, DCB, BRS), ISR definition, imaging modality and definitions (OCT/IVUS criteria), and clinical outcomes (e.g., TLR/TVR, MACE, target lesion failure). Findings were synthesized narratively by thematic domains (mechanisms and vascular healing; neoatherosclerosis; imaging-guided mechanism classification including stent underexpansion; and evidence-based management strategies). Quantitative statements (e.g., prevalence estimates) were reported only when directly attributable to a cited study or high-quality meta-analysis, and observational associations were described as hypothesis-generating where appropriate.

### 2.4. Statistical Analysis

No new statistical analyses or meta-analysis were performed; results are presented as a narrative synthesis of the published literature.

### 2.5. Limitations of the Selection Approach

As a narrative review, this work did not aim for exhaustive retrieval of all eligible studies and did not follow PRISMA methodology, which is designed for systematic reviews. Selection and publication bias cannot be fully excluded, and a formal risk-of-bias appraisal was not undertaken for each included study; to improve transparency and reporting quality, the structure and content were aligned with published recommendations for narrative reviews (including clarity of rationale, transparency of literature identification, and balanced presentation of evidence).

### 2.6. AI Tools

During the preparation of this manuscript, the authors used ChatGPT (GPT-5.2) to support specific tasks (e.g., language editing and restructuring of text). All AI-assisted output was critically reviewed and revised by the authors, who take full responsibility for the final content of this publication.

## 3. ISR Pathophysiology

### 3.1. Neointimal Hyperplasia

The pathophysiology of ISR involves a cascade of events that define the vessel healing process, primarily characterized by tissue proliferation, specifically intimal hyperplasia. This process can result in either vascular healing or pathological progression. Four main interconnected factors contribute to neointimal hyperplasia: endothelial dysfunction, vascular smooth muscle cell (VSMC) transformation, accumulation of monocytes in the subintimal space, and fibroblast migration. Ultimately, the intimal layer of the vascular wall thickens and consists mainly of extracellular matrix components, VSMCs, some fibroblasts, and foam cells. Table 1 shows cellular contributors to coronary ISR, summarizing key effector and inflammatory cell types across early neointimal hyperplasia and late neoatherosclerosis (Table 1).

The initiating factor of this cascade is local mechanical injury to the intimal and medial layers during stent implantation. As a result, VSMCs and the extracellular matrix of the tunica media are exposed to circulating blood components and the stent surface. This mechanical injury, combined with the presence of a foreign body in the vessel, triggers an inflammatory process, which is simultaneously modulated by an anti-inflammatory effect from the drug released by the stent, leading to a slowing of the re-endothelialization process [10]. This inflammatory process involves the expression of adhesion molecules, such as P-selectin, on endothelial cells, which serve as signals for the recruitment of monocytes and the subsequent secretion of pro-inflammatory cytokines, such as tumor necrosis factor-alpha (TNF-α), interleukin-1 beta (IL-1β), interleukin-6 (IL-6), interleukin-8 (IL-8), and growth factors like transforming growth factor β (TGF-β).

The accumulation of monocytes in the subintimal space involves their traversal through the endothelium. The initial steps include the rolling and capture of monocytes attracted by chemokines. These monocytes bind to selectins expressed by activated endothelial cells and are captured through interactions with P-selectin glycoprotein ligand-1 (PSGL-1) and peripheral node addressin (PNAd) [11]. Intercellular adhesion molecule 1 (ICAM-1) and vascular cell adhesion molecule 1 (VCAM-1) facilitate the rolling and adhesion of monocytes, while their transmigration is supported by junctional molecules like junctional adhesion molecule (JAM). Once in the subintimal space, monocytes differentiate into macrophages, increasing endothelial permeability and promoting the formation of foam cells, a process similar to atherosclerosis [12]. The accumulation of lipid-laden foam macrophages can sometimes lead to the formation of a necrotic core, predominantly located in the area of stent implantation [13]. An increase in the number of monocytes and eosinophils three months after PCI is considered a predictive indicator for late ISR following the implantation of a DES [14]. It is well known that mast cells are involved in the process of atherosclerosis and are found at the site of vascular injury. The exact role of mast cells in this process is not yet fully understood, but mast cells release many pro-angiogenic factors, such as TGF-β, TNF-α, IL-8, heparin, and other proteases. They also increase vascular permeability through the release of histamine, thus participating in the process of neointimal hyperplasia [15].

Chemokines such as MCP-1 (CCL2), RANTES (CCL5), and fractalkine (CX3CL1) promote the proliferation and migration of vascular smooth muscle cells (VSMCs) [16,17]. To limit inflammation and ISR, future studies should focus on MCP-1/CCR2 axis blocking medication.

Additionally, perivascular adipose tissue (PVAT), which surrounds most blood vessels, plays a role in triggering inflammation after PCI [18]. PVAT is a connective tissue composed of a mix of cell types, including inflammatory cells such as macrophages, lymphocytes, and eosinophils [19]. In the presence of pathological conditions, PVAT experiences an increase in volume, loses its normal functionality, and triggers inflammatory processes in blood vessels, with a predilection for the coronary arteries [20]. In PVAT associated with atherosclerosis, a significant increase in the concentrations of TNF-α, IL-6, IL-1β, and MCP-1 is observed. The inflammatory process of PVAT is exacerbated by risk factors such as obesity and type 2 diabetes mellitus (T2DM), the latter being considered a possible mechanistic link between T2DM and atherosclerosis [21]. The connection between a triad of pathologies—obesity, diabetes mellitus, and endothelial dysfunction—through the inflammatory processes of perivascular adipose tissue, underscores the importance of evaluating its role in phenomena such as neoatherosclerosis induced by PCI [22].

In endothelial cells, nitric oxide plays a key role in vascular tone, anti-inflammatory and antioxidant effects, along with inhibiting vascular smooth muscle cell migration and proliferation.

Endothelial dysfunction represents the inability of endothelial cells to maintain vascular homeostasis. Numerous studies demonstrate that nicotinamide adenine dinucleotide phosphate oxidases are essential for reactive oxygen species production in the vascular system and neointimal tissue formation, specifically in vascular smooth muscle cell proliferation and migration, including in hyperglycemic conditions [23]. Nicotinamide adenine dinucleotide phosphate oxidase expression is induced by growth factors, cytokines, and other factors that can be elevated in pathological conditions, such as those caused by mechanical injury. Several molecules inhibit nicotinamide adenine dinucleotide phosphate oxidases, such as GKT137831, which can reduce atherosclerosis, but there is a lack of clear evidence regarding whether these molecules could be effective in vivo for attenuating vascular complications after stenting [24,25].

The exact causal mechanisms of this dysfunction are unclear, but increased reactive oxygen species production and endothelial nitric oxide synthase uncoupling are key factors. Superoxide can capture nitric oxide to form peroxynitrite, thereby reducing nitric oxide bioavailability. Nitric oxide is inactivated, and its protective effects, such as reducing platelet/leukocyte adhesion and vascular smooth muscle cell growth and migration, are thus limited [26].

Apart from nitric oxide production by endothelial nitric oxide synthase, nitric oxide can also be stored and released from small molecules called nitric oxide donors, such as S-Nitroso-N-acetylpenicillamine and S-nitroso-N-acetylcysteine, which are currently under investigation to determine whether they could be used therapeutically to treat endothelial dysfunction and limit ISR [22].

Beyond lipid-lowering and antithrombotic therapy, pharmacologic approaches that address endothelial dysfunction and impaired NO–sGC–cGMP signaling—an axis that can be downregulated in cardiovascular disease in the setting of inflammation and vascular dysfunction—may represent a complementary strategy. Vericiguat, a soluble guanylate cyclase (sGC) stimulator that increases cGMP production even when NO bioavailability is reduced, is described as improving cardiac and vascular function and reducing fibrosis, with downstream effects that include lowering vascular tone. On this mechanistic basis, it could be hypothesized to mitigate restenosis-prone vascular responses and potentially improve ischemic symptoms, but dedicated clinical studies in ISR cohorts are currently lacking [27].

Reductive stress is the counterpart of oxidative stress and is characterized by an increase in reducing equivalents, nicotinamide adenine dinucleotide, nicotinamide adenine dinucleotide phosphate, and glutathione. Glutathione is significantly increased, contributing to cytotoxicity through S-glutathionylation of proteins, such as hemoglobin, as frequently encountered in type 2 diabetes mellitus-associated microvascular disease [28]. In some cases, reductive stress, in the form of high nicotinamide adenine dinucleotide to nicotinamide adenine dinucleotide oxidized ratios in mitochondria, is also known to promote reactive oxygen species formation at a level that exceeds the reactive oxygen species scavenging capacity of antioxidants [29]. Despite growing evidence that reductive stress is as important as oxidative stress, it has been less investigated, although it may be a mediator of neointimal hyperplasia and vascular remodeling [22]. It is possible that, in the context of ISR, oxidative stress increases, activating pathways leading to excessive glutathione production [30].

Despite monocyte attraction to the injury site and their role in neointimal hyperplasia, vascular smooth muscle cells are still considered the main contributor to neointimal tissue formation. The phenotypic switching of vascular smooth muscle cells from a quiescent contractile state to a proliferative “synthetic” state is the process by which they gain specific functionalities, such as proliferation, migration, and subsequent extracellular matrix synthesis and pro-inflammatory factor production [31]. This manifests at the level of cytoskeletal proteins, through the activation of actin polymerization and the initiation of tyrosine phosphorylation.

Historically, vascular smooth muscle cell phenotypic transitions have been regarded as a bidirectional process, whereby cells can transition from a contractile to a proliferative phenotype and vice versa, depending on signals and conditions within the local environment, such as local injury associated with stent implantation. In this process, smooth muscle progenitor cells play a crucial role in vascular remodeling and can be transformed into diverse phenotypes under the influence of mechanical factors and growth factor stimuli, including the synthetic/proliferative, inflammatory, osteogenic, endocytic, and other phenotypes [32]. Under the influence of macrophage-derived cytokines, migrated vascular smooth muscle cells subsequently undergo apoptosis.

This switching is the result of multiple factors, such as cyclic stretching that stimulates the myocyte-specific enhancer factor 2B pathway [33]. This leads to the activation of nicotinamide adenine dinucleotide phosphate oxidase isoform 1, which contributes to vascular smooth muscle cell phenotype modification by altering cytoskeletal structure and reducing contractile proteins such as calponin-1 and smoothelina-B. Other factors include cytokines and chemokines such as TNF-α, IL-1b, IL-6, IL-8, IL-17, C–C motif chemokine ligand 2 and 7 (CCL2, CCL7), transcription factors such as Kruppel-like factor 4 (KLF4) and runt-related transcription factor 2 (RUNX2), and growth factors such as transforming growth factor β1 (TGF-β1) and platelet-derived growth factor (PDGF) [34].

Transforming growth factor β produced by monocytes, platelets, and other cells at the lesion site acts on vascular smooth muscle cells by activating small mothers against decapentaplegic proteins or small mothers against decapentaplegic-independent pathways, such as phosphatidylinositol 3-kinase/protein kinase B, determining the phenotype change that leads to massive smooth muscle cell growth, stabilizes atherosclerotic plaques, and accelerates neointima formation, resulting in vascular restenosis [34,35]. Transforming growth factor β signaling pathways mediated by activin receptor-like kinase and Smad-dependent pathways have been discovered to be activated in human vascular smooth muscle cells after injury and in pre-implantation hyperplastic venous grafts [36].

Extracellular vesicles released from activated platelets have been shown to trigger interleukin-6 production in vascular smooth muscle cells while concurrently upregulating αIIbβ3 integrin and P-selectin expression, thereby facilitating interactions between smooth muscle cells and circulating monocytes [37]. In parallel, platelet-derived microvesicles can promote endothelial protein C receptor expansion via TGF-β1 signaling, giving rise to a proliferative endothelial phenotype characterized by von Willebrand factor and CD34 expression [38].

Under the influence of TGF-β1, endothelial cells lose their polarity and cell-to-cell adhesion, detach from the endothelial layer, and migrate to surrounding tissues, gaining mesenchymal characteristics [39]. Thus leading to impaired endothelial junction stability and increased vascular permeability. This process is called endothelial-to-mesenchymal transition (endo-MT) and has been implicated in atherosclerosis progression by increasing fibronectin deposition and adhesion molecule expression. Furthermore, endo-MT-derived, fibroblast-like cells contribute to atherosclerotic plaque progression and destabilize atherosclerotic lesions by altering the collagen-matrix metalloproteinase balance [12].

Normal endothelial cells loose anti-thrombogenicity and other normal characteristics and act as a source of smooth muscle-like cells that form the neointima [32]. On the other hand, fibroblast growth factor acts as an antagonist of transforming growth factor β to inhibit contractile marker expression. Fibroblast growth factor signal intensity is inversely proportional to the degree of vascular smooth muscle cell differentiation, while transforming growth factor β signal intensity is directly proportional.

Nesfatin-1 is an adipocytokine associated with hypertension that triggers vascular smooth muscle cell transition to the synthetic state by increasing protein and messenger RNA levels, as well as promoter activities for matrix metalloproteinase 2 and matrix metalloproteinase 9, but reducing levels and promoter activity of peroxisome proliferator-activated receptor γ in vascular smooth muscle cells [40,41].

PDGF has been shown to stimulate vascular smooth muscle cell conversion to the synthetic phenotype using multiple pathways, including phosphatidylinositol 3-kinase/protein kinase B and phospholipase C gamma [42]. Activated platelets that adhere to the endothelial lesion site release growth factors, including PDGF. PDGF is the first growth factor identified as a regulator of vascular smooth muscle cell dedifferentiation. This induces monocyte chemoattractant protein 1 expression and other monocyte chemoattractants [43]. Yohimbine inhibits PDGF-induced vascular smooth muscle cell proliferation, migration, and neointima formation by suppressing the phospholipase C-gamma 1 pathway [44].

Several transcription factors regulate vascular smooth muscle cell conversion. Kruppel-like factor 4 plays an essential role in synthetic transformation by inhibiting vascular smooth muscle cell contractile gene expression [45]. Transforming growth factor β signaling activation, through lipid metabolism, glucose metabolism, and inflammatory cytokines, upregulates runt-related transcription factor 2 and promotes vascular smooth muscle cell osteogenic differentiation, as well as medial and intimal calcification [46].

Recent studies have highlighted the important role of microRNAs in vascular smooth muscle cell phenotype change. For example, miR-182-3p regulates the contractile phenotype by inhibiting myeloid-associated differentiation marker expression, while miR-206 supports the contractile phenotype and forms regulatory circuits with other proteins [47,48]. Several microRNAs play roles in vascular calcification and osteogenic gene regulation.

Vascular smooth muscle cells produce growth factors, enhancing migration and proliferation not only of vascular smooth muscle cells, but also of fibroblasts present in the adventitia [49].

This causes fibroblasts to undergo a transformation into myofibroblasts that contribute to extracellular matrix formation by increasing protein synthesis of molecules such as type III collagen and fibronectin. While endothelial dysfunction and damage are the main drivers behind neointimal hyperplasia, adventitial fibroblasts play the last major role in neointimal hyperplasia.

In restenotic plaques, reparative type III collagen density is significantly increased, while mature type I collagen density is reduced. This suggests a shift toward a more active and less mature extracellular matrix composition, which could contribute to plaque instability and restenosis progression. Because these cells also acquire smooth muscle cell characteristics by producing smooth muscle actin, they can also migrate into the media, further decreasing the vessel lumen diameter.

### 3.2. Neoatherosclerosis

Neoatherosclerosis appears as a late complication (3–7 years after stent application) of PCI and remains a significant complication and potential threat to patient health [50]. Pathology and intravascular imaging data show that neoatherosclerosis can develop within months to years after stent implantation, occurs earlier and more frequently after DES than after BMS, and has been documented even with second-generation DES [51,52].

Neoatherosclerosis is the formation of a new plaque within the stent, with mechanisms thought to occur in a manner similar to native atherosclerosis, but on a shorter time scale. The resulting plaque may calcify with or without a thin fibrous cap that can rupture can promote thrombus development [53].

Traditional coronary risk factors plausibly modulate neoatherosclerosis by converging on endothelial dysfunction, oxidative stress, and sustained inflammatory activation. Hypertension promotes endothelial activation and increased permeability (reduced nitric oxide bioavailability and increased reactive oxygen species), facilitating leukocyte adhesion, macrophage recruitment, and smooth muscle cell migration/proliferation within the neointima; renin–angiotensin signaling further amplifies vascular inflammation through pro-adhesive and pro-inflammatory pathways. Elevated LDL accelerates lipid entry and retention in the neointima, where oxidative modification and macrophage uptake promote foam-cell formation and necrotic-core development [54,55]. Chronic inflammatory diseases (e.g., rheumatoid arthritis) increase systemic cytokine signaling, which can impair endothelial healing and sustain macrophage activation, providing a biological basis for accelerated atherogenesis; randomized evidence that IL-1β pathway inhibition reduces recurrent atherothrombotic events supports the causal relevance of inflammation to plaque biology [56].

The preferred diagnostic and evaluation investigation of neoatherosclerosis is optical coherence tomography (OCT). Neoatherosclerosis has been identified as neointimal hyperplasia containing lipid or calcified plaque in at least 3 consecutive frames or ruptured lipid neointimal hyperplasia [57]. Lipid plaque is defined on OCT as a region with strong signal attenuation and diffuse border, and lipid-rich if it exhibits a lipid angle greater than 90 degrees. Ruptured lipid plaque is defined as a ruptured fibrous cap and intraplaque cavity. Fibrous plaque has weak signal or heterogeneous content and well-defined borders. Thrombosis is defined as an irregular mass of >250 μm attached to the lumen surface or floating within it [58].

According to Chen et al., 512 patients undergoing optical coherence tomography (OCT) before repeat PCI for second-generation DES ISR were evaluated; neoatherosclerosis (defined as lipidic or calcified neointimal hyperplasia in ≥3 consecutive frames, or ruptured lipidic neointimal hyperplasia) was present in 28.5% (146/512). Among cases with neoatherosclerosis, 56.8% were predominantly lipidic, 30.8% predominantly calcified, and 12.3% showed mixed lipidic and calcific features; the primary endpoint was target lesion failure (cardiac death, target-vessel myocardial infarction, definite stent thrombosis, or clinically driven target lesion revascularization) [50].

The incidence of neoatherosclerosis increased progressively with time elapsed since stent implantation: 20% at 1–3 years, 30% at 3–7 years, and 75% at over 7 years. Factors associated with lipid neoatherosclerosis were renal insufficiency (even mild with estimated glomerular filtration rate <60 mL/min/1.73 m^2^), unfavorable lipid profile, and time since stent implantation, while calcified neoatherosclerosis was correlated with severe renal insufficiency (estimated glomerular filtration rate <30 mL/min/1.73 m^2^), female sex, and time since stent implantation [50].

Calcified neoatherosclerosis around the stent structure appeared in a substantial proportion (>5%) after 3 years (several years earlier than intra-stent calcified neoatherosclerosis), while intra-stent calcified neoatherosclerosis appeared more than 5 years after stent implantation. Lipid neoatherosclerosis is associated with poorer outcomes, worse target lesion failure, post-PCI, compared to calcified neoatherosclerosis when the stent is correctly implanted [50].

Neoatherosclerosis in ISR lesions is a time-dependent phenomenon with different time courses after DES versus BMS implantation, with earlier appearance after DES. In the OCT core-lab study cited (107 ISR patients), time since the index stent procedure was the only independent predictor of neoatherosclerosis, and stent underexpansion was a frequent concomitant finding, highlighting the need to systematically assess mechanical contributors to ISR. Because underexpansion is a preventable mechanism of ISR identified by intracoronary imaging (IVUS/OCT), contemporary consensus statements emphasize imaging-guided evaluation to define the mechanism and guide re-intervention strategy [59,60]. Stent underexpansion is a major mechanical substrate for ISR and, in OCT-based series, appears to be highly prevalent irrespective of stent type. In the cited cohort, underexpansion was observed in 48.5% and 61.1% of ISR lesions (across the two stent groups analyzed), despite acknowledged variability in how underexpansion is defined across studies. This has direct therapeutic implications: when underexpansion is identified, treatment should prioritize mechanical optimization with aggressive lesion dilatation, potentially requiring super–high-pressure non-compliant balloons, rather than proceeding directly to additional stent layers. More broadly, these findings reinforce the procedural value of intravascular imaging guidance during the index PCI to achieve adequate stent expansion, as a modifiable factor that may reduce subsequent ISR.

Firstly, the distinct pathological characteristics of lesions based on the type of stent implanted can lead to different outcomes. BMS ISR lesions predominantly exhibit a diffuse, homogeneous type, consisting mainly of smooth muscle cells intertwined with collagen fibers. Newer DES are typically associated with a more localized restenosis pattern, frequently observed at the stent edges, of a layered type, containing proteoglycans, inflammatory cells, and fibrinoids [61,62]. The vascular wall in second-generation DES ISR might exhibit a diminished response to repeated application of anti-inflammatory and anti-proliferative drugs, whether released through DES or DCB., after developing resistance to the therapeutic effects of DES [62]. Neoatherosclerosis occurs at lower rates with second-generation DES than with first-generation DES, unstable characteristics, such as thin-cap fibroatheroma or neointimal rupture, being rarer in second-generation DES [63].

**Figure 1:**
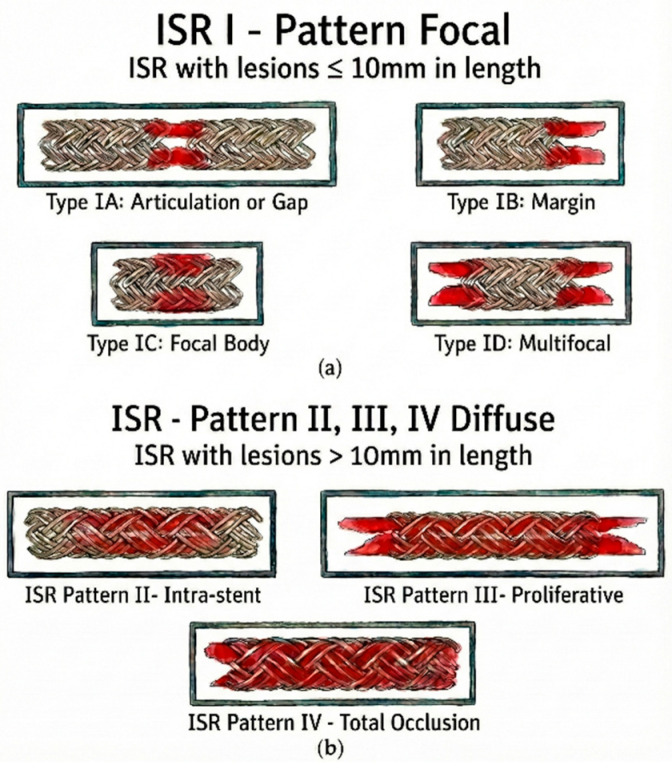
Angiographic patterns of In-Stent Restenosis (ISR) based on the Mehran classification (adapted from an open-access source [64]). (**a**) Pattern I (Focal): Defined as lesions < 10 mm in length. Subtypes include Type IA (Articulation/Gap), Type IB (Margin), Type IC (Focal Body), and Type ID (Multifocal). (**b**) Patterns II–IV (Diffuse): Defined as lesions > 10 mm in length. Subtypes include Pattern II (Intra-stent: confined within the stent), Pattern III (Proliferative: extending beyond the stent margins), and Pattern IV (Total Occlusion).

The schematic delineation portrayed in Figure 1 illustrates the angiographic patterns of ISR [64].

### 3.3. In-Stent Thrombosis

Stent thrombosis after PCI remains a serious multifactorial cause of MI and mortality. Higher thrombotic risk is associated with procedural/lesion complexity such as total stent length >60 mm, bifurcation PCI treated with a two-stent technique, left main PCI, chronic total occlusion PCI, ≥3 lesions treated, and ≥3 stents implanted, as well as with suboptimal PCI results including stent underexpansion, stent malapposition, and residual dissection [60,65].

Antithrombotic factors are also critical: premature discontinuation of antiplatelet therapy is consistently identified as the strongest predictor of stent thrombosis, while high on-treatment platelet reactivity has been associated with early stent thrombosis in contemporary DES cohorts [66]. Disorders affecting platelet number or function can further modify risk: prothrombotic thrombocytosis (e.g., essential thrombocythemia) has been reported in association with post-PCI stent thrombosis, whereas thrombocytopenia (a major high-bleeding-risk criterion when platelet count <100 × 10^9^/L) may necessitate de-escalation or premature interruption of DAPT, indirectly increasing thrombotic vulnerability in the early post-stent period [67,68].

Endothelial cells have an essential role in preventing thrombus formation. When the endothelium is intact, nitric oxide and PGI2 act synergistically to inhibit platelet activation, thus limiting thrombus formation [69]. Increased reactive oxygen species production is shown to be associated with stent thrombosis [70]. When endothelial cells are denuded, the subendothelial extracellular matrix rich in collagen is exposed, leading to platelet attraction and activation.

Subsequent platelet activation and adhesion are mediated by numerous glycoproteins (GPVI, GPIb/V/IX) and their interactions with von Willebrand factor and the collagen-rich extracellular matrix, interconnected with these thrombogenic factors, such as the complement system [71]. Platelets adhere to the lesion site using various mechanisms. These are: collagen recognition from the extracellular matrix by integrin α2β1 and GPVI or attachment through P-selectin glycoprotein ligand-1 and GPIbα [72]. Aggregation occurs through: P-selectin attachment produced by adherent platelets or by their capture by ultra-large von Willebrand factor strings that are produced by endothelial cells under high shear stress, in this case endothelial injury not being necessary [73].

In addition, inflammation can trigger thrombosis and, conversely, thrombosis amplifies inflammation [74]. Platelets are among the first cells that participate in the local inflammatory process. Activated platelets, as mentioned earlier, release PDGF, which favors vascular smooth muscle cell migration and differentiation. Histamine is also involved in neointimal hyperplasia through its pro-inflammatory effect and promotion of vascular smooth muscle cell proliferation [75]. This chronic inflammation, which can be suggested by serum C-reactive protein levels, is caused by the interactions of stent biomaterials with blood, resulting in protein adsorption, platelet activation and adhesion, and activation of the coagulation cascade, as well as inflammation pathways [76].

Regarding the time elapsed from PCI to thrombosis, it can be divided into early, late, and very late. Early in-stent thrombosis occurs in 0.80% of patients treated with PCI and can be subclassified into acute, when it is in the first 24 h, and subacute, between 24 h and 30 days. Late in-stent thrombosis is defined as thrombosis that occurs between 1 and 12 months and occurs in 0.81% of cases. Very late in-stent thrombosis occurs more than 12 months after implantation and is found in 0.77% of cases [77]. Intraprocedural thrombosis is not included in this classification.

In-stent thrombosis, depending on the diagnostic method (which may include clinical, biological, imaging, and anatomopathological data), can be classified into 4 categories: silent occlusion, possible, probable, or definite in-stent thrombosis. Due to the acute nature of the occlusion, it most often manifests as an acute coronary syndrome [78].

**Figure 2:**
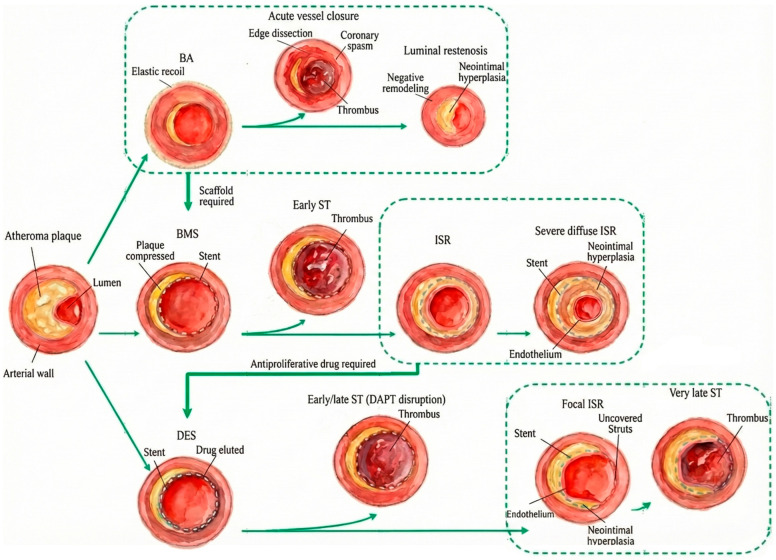
ISR pathophysiology (adapted from an open–access source [80]) Note: BA (Balloon Angioplasty), BMS (Bare-Metal Stent), DES (Drug-Eluting Stent), ST (Stent Thrombosis), ISR (In-Stent Restenosis).

First-generation DES have been associated with higher rates of late and very late thrombosis compared to newer generation DES [79]. It has been observed that thrombosis can occur regardless of the duration of dual antiplatelet therapy, so protection against late DES thrombosis may be due to the delayed endothelialization effect of the coronary lumen by the anti-proliferative drugs released by DES [80]. There is no clear consensus on whether DES are more effective than BMS after 1 year [81]. The lack of a clear advantage of DES could be influenced by the prolonged duration of dual antiplatelet therapy, which typically extends over a minimum period of 6–12 months, in patients who have undergone DES implantation [82]. Figure 2 summarizes the pathophysiology of ISR.

## 4. ISR Clinical Presentation

ISR typically manifests 3 to 12 months after stent angioplasty [82]. ISR continues to be the main cause of target lesion failure after stent implantation, leading to an incidence of approximately 20% of target lesion revascularization at 10 years [83,84]. Target lesion failure is defined by: cardiovascular death, target vessel myocardial infarction and target lesion revascularization. Another clinical endpoint is stent/scaffold thrombosis [85].

The clinical presentation of ISR can be classified as either acute coronary syndrome or non-acute coronary syndrome, and the mode of presentation depends more on patient-related factors than stent-related factors. According to a study that included 909 patients (1077 ISR lesions), acute coronary syndrome is the most frequent mode of presentation of ISR [5]. As shown by more recent studies, acute coronary syndrome was encountered in over 60% of patients, regardless of the type of stent used, and among these, 44% presented with unstable angina, 44% with non-ST-elevation myocardial infarction, and 12% with ST-elevation myocardial infarction. Despite early revascularization, 6-month mortality remains very high in these patients [82]. Approximately 40% present as non-acute coronary syndrome, of which 86.3% present with recurrent stable angina and 13.7% with silent myocardial ischemia [86]. Patients who present with ISR are under pharmacological treatment, so it is very unlikely that these patients would complain of atypical symptoms and be ignored.

In a retrospective analysis of 11,666 patients treated with PCI, patients who presented with ISR after DES implantation manifested signs and symptoms of unstable angina pectoris more frequently compared to those who had de novo stenosis (61% vs. 45%), and the major adverse cardiovascular event rate was 17% (compared to 10%). While myocardial infarction presentation was rarer in the ISR group (6% vs. 14%) [87].

ISR can trigger an acute coronary syndrome as a result of a superimposed thrombus, aggressive hyperplasia patterns, or both. The culprit areas of ISR are rich in macrophage cells, neovascularization, and tissue factor that can trigger an acute coronary syndrome, while thrombus was documented more frequently in acute coronary syndrome, as opposed to non-acute coronary syndrome ISR. In contrast, a diffuse restenotic lesion that limits flow itself could be the nidus for thrombus, which can also lead to an acute presentation [5].

The mode of presentation in ISR in first-generation DES cannot be distinguished from that of BMS, even though DES have been associated with a lower rate of ISR and present a more focused angiographic pattern [5,86].

The clinical presentation modality of acute coronary syndrome lesions is not associated with differences in the angiographic appearance of ISR lesions, the lesions being similar between acute coronary syndrome and non-acute coronary syndrome. Also, there were no significant differences between them related to disease burden, vessels affected by ISR, and ISR location. In contrast, clinical presentation influences the prognosis of the patient with ISR. Major adverse cardiac events, defined as a composite of cardiac death, myocardial infarction, and clinically indicated target lesion revascularization, were used as the primary endpoint. Acute coronary syndrome presentations caused by ISR had a higher incidence of major adverse cardiac events and all-cause mortality at 1 year, compared to non-acute coronary syndrome but also to patients with de novo lesions, independent of patient comorbidities [5,86,88]. Therefore, it is recommended to identify individuals at risk of acute coronary syndrome presentation and to closely monitor ISR patients who have presented with an acute coronary syndrome.

Intravascular imaging techniques such as OCT or intravascular ultrasound (IVUS) have significantly contributed to deepening the understanding of the relationship between specific plaque characteristics and the probability of ISR [89]. Recent data suggest that OCT diagnosing accuracy of this ISR is comparable to that of angiography [90,91].

For example, factors highlighted by OCT, such as inadequate initial stent expansion, the presence of significant neointimal hyperplasia or calcifications around the stent, and the presence of multiple stent layers, can contribute to suboptimal new stent expansion. This suboptimal expansion has been associated with poorer long-term outcomes, including an increased risk of myocardial infarction and the need for repeat revascularization procedures within two years [92]. OCT also allows the classification of neointimal hyperplasia lesions into 6 types with different clinical impact: homogeneous high-intensity tissue (type I), heterogeneous tissue with signal attenuation (type II), speckled heterogeneous tissue (type III), heterogeneous tissue containing poorly delineated region with invisible strut (type IV), heterogeneous tissue containing sharply delineated low-intensity region (type V), or bright protruding tissue with an irregular surface (type VI) [93]. Nonetheless, the current body of evidence remains insufficient to substantiate the efficacy of intravascular imaging because of ISR relatively small incidence and the unclear use of the information provided by intravascular imaging [94].

## 5. Risk Factors for ISR

A study showed a higher percentage of events in patients over 75 years of age with BMS compared to patients with DES (16% vs. 12%) [95]. Another factor increasing cardiovascular risk is related to sex. Men have traditionally been considered at a higher risk compared to women; however, recent years have shown that the incidence of cardiovascular events in women has been underestimated, with nonspecific symptoms complicating the diagnosis in such cases. Moreover, the majority of studies have underrepresented women in their populations, despite the higher prevalence of associated risk factors, such as diabetes, in women. Women tend to have an increased cardiovascular risk through the development of comorbidities like dyslipidemia or diabetes, especially due to hormonal changes brought about by menopause. In the literature, ACS occurs 3–4 times more frequently in men than in women under the age of 60, but above 75 years, women represent the majority of patients. In other words, cardiovascular mortality is similar between the sexes starting from the 7th decade of life [96]. On the other hand, studies investigating the risk of major cardiovascular events did not find sex to be an independent predictor in patients treated with coronary stenting, a result consistent with ours [77,97].

Hypertension (HTA) is often the most frequent risk factor in patients with heart disease. There is also a correlation between systolic blood pressure (SBP) and the risk of cardiovascular events [98]. It is associated with other cardiovascular comorbidities in metabolic syndrome, such as obesity, diabetes, hyperuricemia, dyslipidemia, and sleep apnea syndrome.

A retrospective study observed a connection between ISR and HTA, with patients who had normal blood pressure values at the time of stent implantation having a 24% lower risk of developing ISR [99]. Among stented patients, the prevalence of hypertension, smoking, and type 2 diabetes was higher in the ISR group on univariate analysis and these factors remained independently associated with ISR in multivariable logistic regression (HTA, smoking, and type 2 diabetes all retained statistical significance) [100].

Smoking has a strong prothrombotic effect secondary to vascular dysfunction induced by nicotine consumption. The reduction in nitric oxide concentration, leading to increased adhesiveness of adhesion molecules at the blood vessel level, results in endothelial dysfunction. The aggregation of plasma compounds creates a procoagulant and proinflammatory local environment, perpetuating the pathophysiological process initiated by smoking [101]. Smoking not only has a direct effect but also an indirect one by promoting endothelial remodeling and increasing thrombotic incidents. Smoking has been described as a predictor of ISR by increasing neointimal hyperplasia and the rate of malposition of “struts” [102].

Dyslipidemia, represented by increased blood lipid and cholesterol levels, particularly LDL (low-density lipoprotein) cholesterol, is directly involved in increasing cardiovascular risk through accumulation in the blood vessels. Its deposition in the blood vessel following erosion in the context of chronic inflammatory syndrome, along with plasma compounds, leads over time to the formation of atherosclerotic plaques.

A study that included approximately 180,000 patients, divided into three risk groups, found that in the highest risk group, where the target LDL-c was <70 mg/dL, this was achieved in only 25.4% of the patients. This may be due to poor adherence to lipid-lowering treatment, treatment inefficacy, lack of symptoms, and insufficient patient education, leading to a less serious view of the pathology. Additionally, physician inertia in escalating lipid-lowering therapy to reach new target values has also been observed [103].

HDL (high-density lipoprotein) cholesterol is known to be the protective fraction of cholesterol, responsible for transporting cholesterol to the liver for elimination. Thus, low HDL cholesterol levels are associated with a higher risk of ACS and unfavorable outcomes post-ACS [104]. A study that analyzed the LDL-c/HDL-c ratio in 216 patients after stent implantation showed that the LDL-c/HDL-c ratio was higher in the ISR group than in the control group, with LDL-c/HDL-c ratio and diabetes mellitus identified as independent risk factors for ISR [105]. Another study, which included 368 patients with diabetes mellitus, found a significant association between VLDL-c levels, triglycerides, and uric acid and the risk and severity of ISR [106].

Obesity is one of the most important cardiovascular risk factors and is widespread. This condition is often associated with HTA, diabetes, and the development of sleep apnea syndrome or Pickwick syndrome. Globally, the prevalence of obesity has increased from 8.8% in 1990 to 18.5% in 2022 in women and from 4.8% to 14.0% in men. Worryingly, the number of affected children is also increasing [107].

The degree of obesity is quantified by calculating BMI or measuring waist circumference, which classifies the patient into a certain risk group. The score is adjusted based on ethnicity. In a large contemporary cohort study by Jones et al., ISR was associated with a higher 1-year risk of major adverse cardiovascular events (MACE), defined in that study as a composite of all-cause death, myocardial infarction, and target-vessel revascularization, and this excess risk was consistent across BMI categories (i.e., irrespective of BMI), being largely driven by target-vessel revascularization [108].

Diabetes mellitus is a well-known risk factor closely linked to the atherosclerotic process, one of the mechanisms being the formation of Advanced Glycation End Products (AGEs), which induce endothelial dysfunction and oxidative stress. A recent meta-analysis that included 20 randomized controlled trials concluded that patients with diabetes exhibit a significantly higher risk of primary restenosis following PCI compared to those without diabetes. This finding suggests that diabetes is an independent risk factor for ISR regardless of glycemia, duration of antiplatelet therapy, or the specific vessel treated [109]. Conversely, the exact mechanism through which diabetes promotes ISR is still unclear.

Patients with hyperglycemia, such as those with type 2 diabetes, have chronic inflammation, with elevated serum levels of inflammatory cytokines such as IL-6 [110] and upregulation of the expression of cellular adhesion molecules (VCAM-1, ICAM-1, and E-selectin) and a chemokine (MCP-1/CCL2). It has been shown that vascular complications in T2DM, as well as VSMC proliferation at the intimal level, are mediated by IL-6 and its signaling pathways [111]. For patients with diabetes, endothelial cell denudation may have an aggravated effect due to reduced bioavailability of NO and increased endothelial NOX activity in the presence of hyperglycemia [112].

The association between CKD and cardiovascular diseases leads to increased mortality and the occurrence of major cardiovascular events, and the risk of restenosis in stents is much higher. Additionally, the presence of proteinuria and/or reduced glomerular filtration rate (GFR) significantly increases the prevalence of cardiovascular diseases [113].

The presence of chronic kidney disease (CKD) is associated with a higher risk of restenosis and all-cause mortality following coronary interventions, with the greatest risk observed in patients receiving hemodialysis. In addition, patients with CKD who develop in-stent restenosis (ISR) more frequently present with acute coronary syndromes. In a cohort study of 1376 patients treated with DCB angioplasty, 639 patients had CKD (defined as an estimated glomerular filtration rate [eGFR] <60 mL/min/1.73 m^2^) and 737 had preserved renal function. After multivariable adjustment, patients with severe CKD (eGFR 15–29 mL/min/1.73 m^2^) and those with end-stage renal disease (ESRD; eGFR < 15 mL/min/1.73 m^2^) exhibited a significantly higher risk of adverse clinical events compared with patients with preserved renal function [114].

Furthermore, in a patient with significant renal impairment, the administration of contrast agent (necessary for PCI) may increase the risk of worsening kidney disease. Consequently, a comprehensive assessment of renal function is essential before PCI.

Carotid artery examination is an indirect indicator for assessing generalized atherosclerosis, easily reproducible, encompassing aortic, coronary, and peripheral atheromatosis. Even non-hemodynamically significant carotid artery atherosclerosis may be an important indicator of systemic disease.

Among patients with STEMI, a special category was those treated with thrombolysis in the territory, as the time to reach the PCI center was long, which contributed to a better prognosis regarding ISR. The incidence of thrombolyzed STEMI patients was 18%, i.e., 19 cases out of the studied cohort.

Atrial fibrillation (AF) in the context of AMI is associated with unfavorable clinical outcomes, likely due to the increased risk of complications such as heart failure and stroke, as well as the heightened risk of death in-hospital and readmission within 30 days [115].

AF usually develops secondary to complications in STEMI. However, AF is an independent predictor of short-term and long-term adverse outcomes in this group of patients, regardless of the reperfusion strategy. AF is associated with a significant risk of coronary embolization [116], and it was observed that it is associated with an increased risk of ISR in other locations [117]. Another study assessing risk factors for in-stent thrombosis did not find AF as a predictive factor [77].

One study showed that the presentation of patients in Killip class III-IV is an independent predictor for in-stent thrombosis [118]. Furthermore, patients with reduced EF were found to have an increased risk of ISR and in-stent thrombosis [119,120].

It is documented that certain coronary arteries are more prone to restenosis than others (e.g., LAD). Lesion characteristics, such as proximal ISR location or involvement of the left anterior descending artery, were not associated with ACS presentation [86].

Compared with BMS, first-generation DES reduce classic ISR driven by neointimal hyperplasia, but they also delay re-endothelialization relative to BMS [6]. OCT data shows that neoatherosclerosis is time-dependent and tends to appear earlier in DES-ISR than in BMS-ISR, because neoatherosclerosis is a distinct later substrate of stent failure from early neointimal hyperplasia [59]. It was observed that a greater number of stents implanted in the same patient is associated with a significantly higher risk of ISR [112,119]. This observation may be related to the increased stent material surface, which may contribute to remodeling and impede blood flow. The presence of an implanted stent in a coronary artery at the time of the acute event is associated with a higher percentage of ISR lesions.

The characteristics of the chosen stent that depend on the vessel anatomy are relevant: the longer and thinner the stent, the higher the risk of restenosis. Vascular caliber represents a predisposition factor for restenosis. Treatment of thin vessels leads to poor evolution and a higher degree of restenosis [100]. Small vessels have less space for neointimal accumulation, so even a small difference in “late loss” can be significant. Smaller stents (with a diameter <3 mm) and longer stents (>20 mm) were associated with higher ISR, with statistically significant differences between groups [121,122]. Pre-PCI and post-PCI angiographic minimum lumen diameters (MLD) were narrower, and the length of ISR was greater in lesions that underwent target lesion failure compared to those that did not [50].

Significantly increased restenosis rates are observed in cases characterized by a higher number of lesions and more complex lesions, such as those involving small vessels, extensive lesions, or bifurcation lesions.

Simultaneous thrombosis in multiple coronary arteries is an unusual angiographic finding in STEMI but is associated with higher mortality [119]. Patients with additional lesions identified on follow-up angiography more frequently have a history of vascular atherosclerotic disease and may exhibit a higher risk of subsequent vascular events. Average time to restenosis has been observed to be modestly shorter. The literature addressing this specific association remains limited [100].

Incomplete restoration of coronary perfusion (TIMI flow ≤ 2) together with greater residual narrowing can sustain disturbed flow patterns, reduce endothelial healing, and favor repeated luminal loss. In reported cohorts, achievement of TIMI grade 3 flow has been less frequent among patients who develop ISR, and intra-procedural adverse events have occurred at a significantly higher rate in this group [123].

Regarding the prognosis of BCV, implicitly ACS, both short-term and especially long-term, another parameter used in the studied cohort was antithrombotic treatment. Antithrombotic treatment, DAP or DAP plus anticoagulant, plays an essential role in preventing ISR [124]. The chosen P2Y12 inhibitor was not an independent risk factor for ISR [100,119].

Statin therapy is part of the elective treatment for AMI. The pleiotropic effects of statins include improving endothelial dysfunction, increasing the bioavailability of nitric oxide, antioxidant properties, inhibiting inflammatory responses, and stabilizing atherosclerotic plaques. Many of the pleiotropic effects of statins function independently of LDL cholesterol reduction, are weakly or not correlated with LDL-cholesterol changes, occur rapidly, and are rapidly reversible when the treatment is stopped [125]. Acute coronary lesions in patients treated with therapeutic statin therapy, compared to those not on statin treatment with elevated LDL, tend to be longer, more calcified, with increased tortuosity and angulation of vessels, as well as from a stent or bypass graft mechanism. These clinical findings are likely the result of altering the natural history of plaque pathophysiology observed with statin therapy [126].

## 6. Management

Intravascular imaging—using OCT or IVUS—is central to identifying the true mechanisms behind ISR and stent thrombosis. These techniques can distinguish un-derexpansion, calcific neoatherosclerosis, stent fracture, or homogeneous neointimal proliferation, allowing treatment to be tailored to the specific failure mechanism [127].

Compared with IVUS, OCT provides substantially higher spatial resolution (≈10–20 μm vs. ≈100–150 μm) and therefore offers more detailed visualization of stent struts, malapposition/edge pathology, and neointimal coverage. Conversely, OCT has limited tissue penetration (≈1–2 mm) relative to IVUS (≈4–8 mm), which can prevent complete visualization of the full vessel wall and external elastic lamina—particularly in large proximal vessels—whereas IVUS can assess deeper vessel layers (including adventitia) and is commonly used for sizing/optimization strategies. Because OCT requires transient blood clearance with contrast injection, modality selection in large proximal segments (e.g., LMCA/proximal LAD) should be individualized based on vessel size, ostial anatomy, and contrast load [128,129,130].

Neither modality has yet proven outcome superiority in randomized trials. In a substudy of ILUMIEN IV, OCT-guided PCI in complex lesions resulted in more rigorous lesion preparation, larger and longer stent implantation, and greater stent expansion than angiography guidance. These improvements led to a 37% reduction in serious MACE (cardiac death, target-vessel MI, and stent thrombosis) at 2 years. Despite these benefits, OCT guidance did not significantly reduce target-vessel failure. Future work should clarify which imaging-derived features guide optimal device selection—such as DCB versus DES—and establish procedural success criteria, particularly given the multiple mechanisms un-derlying ISR [131].

Non-invasive coronary physiology assessment is evolving beyond intravascular imaging, with CCTA-derived FFRCT (increasingly implemented via machine-learning approaches) enabling functional interrogation from CT datasets. In patients with prior coronary stent implantation, preliminary observational data suggest feasibility for physiological assessment of suspected ISR: in a retrospective cohort with paired invasive FFR, ML-based FFRCT demonstrated good correlation with invasive FFR and approximately 86% accuracy for detecting hemodynamically significant ISR. As FFRCT has been associated with improved specificity of CCTA and fewer “lesion-free” invasive angiograms in broader CAD diagnostic pathways, these early stent-specific data support the concept of expanding non-invasive triage (CCTA + FFRCT) to reduce repeat invasive angiography in selected stented patients, while recognizing ongoing constraints from stent artefacts and the need for prospective validation in larger, higher-risk cohorts [132].

Bioresorbable scaffolds (BRS) were initially introduced with the aim of restoring physiological vascular function while avoiding the persistent adverse effects associated with permanent metallic stents.

Predictive models and risk-stratification tools are being developed to identify candidates most likely to benefit from BRS and to reduce adverse event rates through informed procedural planning.

Alongside polymer-based devices, magnesium-based scaffolds have emerged as a viable next-generation alternative. Systems such as the DREAMS 3G platform offer accelerated degradation while maintaining sufficient radial support during early vas-cular healing, potentially mitigating risks of late inflammation, neoatherosclerosis, and restenosis [133].

Evidence from contemporary randomized trials, including ABSORB IV, suggests that when BRS are deployed in appropriately selected patients and with optimized procedural protocols, clinical outcomes approach those of contemporary DES. The trajectory of BRS development now rests on continued material innovation, high-quality clinical validation, and personalized treatment strategies.

The field is moving toward improved scaffold materials, thinner struts, procedural optimization, and long-term studies to validate a true “leave-nothing-behind” strategy. Ongoing research on new DCB and DES for ISR highlights that meticulous lesion preparation remains fundamental, and future trials should consider randomization after imaging-guided preparation to match therapy to lesion biology.

Managing ISR requires an approach tailored to both the mechanism of failure and the lesion’s morphology. Plain balloon angioplasty is no longer used as a definitive treatment because of high recurrence, but non-compliant and ultra–high-pressure balloons remain important for correcting stent underexpansion and for lesion preparation before definitive therapy. Cutting and scoring balloons improve neointimal modification and can enhance the effect of DCB, particularly in DES-ISR, by providing controlled plaque incision and reducing balloon slip-page.

Ablative therapies—including rotational atherectomy, “rotatripsy,” and excimer laser coronary atherectomy—may be used selectively for heavily calcified neoathero-sclerosis or resistant ISR, mainly as adjuncts to facilitate DCB or DES delivery. Evidence remains limited, and these techniques are generally reserved for complex or recurrent cases.

DCBs offer antiproliferative therapy without adding a new stent layer, making them especially useful for ISR with multiple stent layers or malapposi-tion. Paclitaxel-coated balloons currently have the strongest evidence base, though emerging sirolimus-based and biolimus-based balloons show promise in early studies. DCBs are guideline-recommended in Europe for ISR treatment.

Repeat DES implantation—particularly with modern everolimus-eluting stents—remains an effective option, often providing superior angiographic results compared with DCBs, though with a higher risk of adverse events than DES use in de novo lesions. Data on whether switching the antiproliferative drug improves outcomes are mixed [134].

Intravascular brachytherapy has re-emerged for refractory DES-ISR by inhibiting neointimal proliferation, although access is limited and robust randomized data are lacking [135].

Intravascular lithotripsy may assist in treating calcified, undilatable ISR by modi-fying deep calcium to permit adequate stent expansion, but current evidence is obser-vational, and its use in ISR remains off-label.

Overall, contemporary ISR management relies on precise lesion preparation and careful selection of DCB, DES, IVBT, or adjunctive ablative therapies according to the underlying mechanism of restenosis.

## 7. Conclusions and Take-Home Messages

ISR remains a frequent and clinically meaningful cause of recurrent angina, acute coronary syndromes, and repeat revascularization, even in the contemporary era of second-generation DES. Importantly, restenosis risk does not end after the first year but accumulates over time and with repeat interventions, and PCI performed for ISR is consistently associated with worse outcomes than PCI for de novo coronary lesions.

From a clinical perspective, ISR should be viewed as a heterogeneous, mechanism-driven condition rather than a uniform entity. Early ISR is predominantly related to neointimal hyperplasia following vascular injury, whereas late ISR often reflects in-stent neoatherosclerosis and may present with unstable clinical syndromes. Patient comorbidities—particularly diabetes, chronic kidney disease, persistent dyslipidemia, hypertension, and smoking—together with lesion complexity, long or multiple stent segments, and inadequate stent expansion, substantially influence both the likelihood and the clinical expression of ISR.

The most important practical implication for clinicians is to prioritize prevention and mechanism-directed management. Optimal stent implantation at the index procedure—ensuring appropriate sizing and full expansion and using intravascular imaging when angiographic uncertainty exists—represents the most effective strategy to reduce future ISR. When restenosis occurs, intravascular imaging with IVUS or OCT is central to identifying the dominant mechanism (e.g., underexpansion, neoatherosclerosis, or tissue proliferation) and should guide the choice between repeat DES implantation, drug-coated balloon angioplasty, or adjunctive lesion-modifying techniques.

Beyond reintervention, aggressive secondary prevention is essential. Strict adherence to antithrombotic therapy, intensive lipid lowering, and comprehensive control of cardiovascular risk factors are critical to limiting recurrent stent failure and late adverse events. Finally, emerging mechanistic insights into inflammatory, metabolic, and redox pathways highlight promising future therapeutic directions; however, their clinical application will depend on prospective, adequately powered studies and standardized phenotyping of ISR in routine practice.

## Figures and Tables

**Table 1:** Cellular contributors to coronary ISR.

Cell Type	Where/When It Matters Most	Primary Contributions to ISR Biology (Shortened)
Endothelial cells (ECs)	Early after PCI; also chronic dysfunction/delayed healing (esp. DES)	Delayed re-endothelialization; pro-inflammatory surface
Endothelial progenitor cells (EPCs)	Repair phase; therapy concepts (EPC-capture/pro-healing strategies)	Endothelial repair support
Vascular smooth muscle cells (VSMCs)	Core effector in early ISR (neointimal hyperplasia)	Phenotypic switch with proliferation/migration
Smooth muscle progenitor cells	Early remodeling/injury response	VSMC-like remodeling cell source
Monocytes	Early recruitment; also late neoatherosclerosis	Recruitment and macrophage precursor
Macrophages	Early (inflammatory amplification) and late (neoatherosclerosis)	Inflammation; foam-cell formation
Foam cells (lipid-laden macrophages)	Late ISR (neoatherosclerosis); can appear in neointima	In-stent plaque buildup
Neutrophils	Early after injury	Early inflammatory amplification
Lymphocytes (T/B cells; broad category)	PVAT-driven inflammation; chronic vascular inflammation	Chronic immune signaling
Eosinophils	Signal of inflammatory state (association reported)	Inflammatory association signal
Mast cells	Injury site; neointimal hyperplasia (supporting role)	Degranulation-driven permeability/inflammation
Adventitial fibroblasts → myofibroblasts	Neointimal hyperplasia/fibrosis component	ECM deposition and fibrosis
Platelets	Periprocedural and ongoing (thrombo-inflammation)	Growth-factor release; thrombo-inflammation
PVAT adipocytes (and local stromal cells)	Systemic-risk amplifier (obesity/T2DM context)	Perivascular inflammatory milieu

Note: ECs = endothelial cells, EPCs = endothelial progenitor cells, VSMCs = vascular smooth muscle cells, PCI = percutaneous coronary intervention, DES = drug-eluting stent, ISR = in-stent restenosis, ECM = extracellular matrix, PVAT = perivascular adipose tissue, T2DM = type 2 diabetes mellitus, PDGF = platelet-derived growth factor, TGF-β = transforming growth factor beta, Smad = “small mothers against decapentaplegic” proteins, IL-1β = interleukin-1 beta, IL-6 = interleukin-6, IL-8 = interleukin-8, TNF-α = tumor necrosis factor alpha, ICAM-1 = intercellular adhesion molecule-1, VCAM-1 = vascular cell adhesion molecule-1, EndMT = endothelial-to-mesenchymal transition, MCP-1 = monocyte chemoattractant protein-1, CCL2 = C-C motif chemokine ligand 2 (same as MCP-1), CCL5 = C-C motif chemokine ligand 5, CX3CL1 = CX3C chemokine ligand 1 (fractalkine), NO = nitric oxide, NOX = NADPH oxidase, ROS = reactive oxygen species, oxLDL = oxidized low-density lipoprotein, vWF = von Willebrand factor.

## Data Availability

The original contributions presented in this study are included in the article. Further inquiries can be directed to the corresponding author.
